# Case of Removal of the Inverted Uterus

**Published:** 1856-10

**Authors:** 


### *Case of Removal of the Inverted Uterus.* By C. G. PUTNAM, M. D., Boston.

[Read before the Boston Society for Medical Improvement, February 11th, 1856, and communicated for the Boston Medical and Surgical Journal.]

Dr. Putnam gave a history of three cases of removal of inverted uterus, under the care of Dr. Channing and himself. The specimens were exhibited at a previous meeting.

It is now three years since the operations were done, and during this interval we have carefully watched the result. One of the patients was for some months subject to leucorrhœal discharges, and in another there has been an occasional approach to something like menstruation; but they are at present in excellent health and spirits, illustrating the observation of M. A. Petit, that the uterus belongs less to the individual than to the species, and proving that nature can support the loss without material disturbance in the harmony of her functions.

The first was that of a young woman, 20 years of age, with her second child. On application to Dr. Channing, she stated that "dreadful" pain attended the extraction of the placenta, and that the "flowing" was excessive. She was able to nurse her child for three months, though flowing more or less all the time. Immediately upon the suspension of nursing, the hemorrhage became incessant; and when visited, twelve months after childbirth, she was bloodless, anasarcous, and hardly able to move about. He attempted, under the influence of ether, to re-invert the uterus; but failing in this, the ligature of cord was applied, and the ends brought through so that the pressure could be graduated by a screw. The ligature came off on the eleventh day; but in this, as in the other two cases, whenever the pressure was carried beyond a certain point, there ensued vomiting, faintness, depression of pulse and other symptoms of strangulation, which made it necessary to relax it. Her recovery was perfect.

Case II.—The result of this case was not so fortunate. The patient recovered from the effect of the operation, but died from the effects of ill-timed exertion, in the same manner as, after an exhausting hemorrhage, death sometimes follows the mere rising up in bed. A young woman, originally of healthy constitution æt. 25. She had had two confinements within three years. The first time had twins, and was much enfeebled by nursing both. The third child she nursed nearly nine months, and was "pretty well," though frequently "flowing." When she ceased nursing, menstruation recurred at short intervals, and very copiously, and she began to suffer palpitation, throbbing in head, faintness and dyspnœa, on any exertion. It was evident that these symptoms were sympathetic with some uterine lesion, and upon further inquiry it appeared that at the time of delivery, though not aware of any extraordinary pain, hemorrhage, or faintness, yet she never "felt quite right" about the pelvis. During the first week sat up in bed, and moved about the bed more freely than usual. On the eighth day, having got out of bed to evacuate the bowels, she felt something protruding from the external organs, considerably larger than an orange. She suffered much distress until it was replaced in the vagina; and though it never again appeared externally, she was occasionally obliged to press it upward, in order to relieve a painful sense of pressure. The local uneasiness gradually diminished, and she continued to nurse her child till it died at the ninth month. Immediately upon weaning, she began to "flow" almost constantly, but was able to attend to her household duties for eight months, when she suffered so much from faintness that she was compelled to remain in bed.

The most prominent symptoms at this time, when I was consulted, were palpitation, throbbing in the head, dyspnœa on motion, urgent thirst. She was exceedingly pale; pulse 120, feeble; tongue white. On examination, a tumor was felt high up the vagina, apparently about two inches in length, an inch and a half in thickness, and about two inches in breadth. The os uteri soft and distensible, and so nearly effaced that it might readily have been mistaken for a fold of the vagina encircling the upper part of the tumor. This sulcus, about two thirds of an inch deep, could be traced all round the circle, and at no point could the finger or sound be passed further. The color was a deep strawberry red.

She had just finished a menstrual period, and I decided to do the operation at once, so that it might be completed before the recurrence of another. The ligature was applied on the upper portion of the tumor, (it could not be called a neck, for there was less narrowing than one would have expected,) the ends being passed through a canula, and made fast to a button moved by a screw.

The first constriction was followed in about four minutes by pain, failure of the pulse, which dropped from 120 to 90, coldness of the surface, vomiting, and other symptoms of strangulation. It was immediately relaxed, and the pain subdued by opiates and inhalation of ether. During the night, vomited twice. Slept at intervals, opiate repeated. During the next day had copious, serous discharge from vagina. The tumor tense, not tender to touch. Pulse 112. Skin soft. The ligature again tightened, causing very severe pain, which recurred in paroxysms. Opiates and ether repeated.

It will not be necessary to give the occurrence of each day, but I will merely say that the ligature was tightened every twenty-four hours as much as the patient could endure. The excretion of serum continued quite freely —at last attended with fœtor. On the eighth day the tumor was less tense, but as it still seemed not to be entirely detached, no efforts were made to remove it.

Up to the ninth day, she was evidently improving in spirits, appetite and strength. She had slept well the previous night, and in the morning, without asking leave, had her clothes changed and her bed made; and when visited soon afterward, she was very languid, unwilling to move, not faint, but "terribly tired." From this state of depression she never rose; her slight stock of strength had been entirely wasted by this unnecessary exertion, and she died in three days afterward.

On examination after death, the tumor was found to be detached—hanging by a few shreds only. The pelvic organs were healthy, cicatrization perfect, the strangulation of the tumor having thus been effected without inducing peritonitis, and with comparatively slight constitutional irritation.

Case III.—A young woman, æt. 23. Second confinement. She stated to Dr. Channing that she had unusual pain and hemorrhage during the delivery of the placenta. Was unable to nurse the child on account of deficient nipples, and flowing continued for a year, almost without interval. The exhaustion became extreme, and she seemed to be failing rapidly.

In this, as in the other cases, an ineffectual attempt was made to re-invert the uterus before applying the ligature. She was unusually sensitive, and it was sometimes necessary to change the pressure of the ligature three or four times in the course of a day. The tumor was separated in about a fortnight.

From the symptoms which attended these three cases, it is probable that inversion occurred at the time of delivery. There is reason to believe, however, that it may take place gradually, and some evidence is offered to show that it may occur considerable time after an apparently natural delivery. A case is quoted by Dailliez, in which inversion had taken place in a second confinement, and was successfully reduced. A third labor was natural, and on the fourth every precaution was taken to avoid the accident at the time, and during her convalescence; but on the thirteenth day, while sitting up for an evacuation, the inverted uterus, of the size of a fœtal head, suddenly protruded externally.

Levret, in his treatise of polypus, cites the case of a woman who applied to a surgeon on account of certain uncomfortable sensations that came on five years after the birth of her last child, then ten years of age. A pessary was applied, on the presumption that the symptoms were caused by prolapsus, and she was still wearing it, when, five years later, she consulted Levret, who detected inversion of the uterus. In the former case, the attendant, with the apprehension of the accident before him, was satisfied that no inversion existed previously to the thirteenth day; and, in the latter, five years of uninterrupted health had intervened between the parturition and the symptoms of inversion.

A similar case to this may be found in the Med. Times and Gazette, 1855. The patient had been in good health, with the exception of pain in the back, for two and a half years after the birth of her last child. For the two following years had incessant hemorrhage, and on examination she was found to have inversion, which was successfully operated on by Mr. Teale, by means of double ligatures. Mr. Teale's remarks apply equally well to both cases: "That the inversion, if it occurred at the time of the last labor, must have been unattended at the time by any of the symptoms indicative of the accident, and must have remained two and a half years without causing inconvenience, which is highly improbable. On the other hand, if it occurred two and a half years ago, when the symptoms indicative of it commenced, it is remarkable that it should have arisen independently of polypus or pregnancy."

We can readily imagine how easily a flaccid uterus may be inverted by a moderate force applied from without or within. The causes most commonly assigned are a hurried delivery in the erect posture, and traction on the placenta while yet attached. The uterus also may contract sufficiently to detach the placenta, but not with energy enough to expel it; and if traction were made while the placenta was thus enclosed in the cavity, a vacuum might be produced, and the uterus thereby drawn downward. I am not aware that inversion has ever been attributed to such a cause, but I should consider it unsafe to extract by the funis without at the same time raising up an edge of the placenta whenever there was any undue resistance to its descent.

Besides the effect of mechanical force, it has been maintained that inversion may be spontaneous; that the uterus, on account of its previous condition, and by its own action at the moment, inverts itself. Mr. Crosse "does not believe that inversion can ever be spontaneous in this sense," and that some mechanical force is at least necessary to commence it. This could not be disputed if the uterus were an inorganic sac; but when we consider its irregular spasmodic action—sometimes annular, sometimes longitudinal, sometimes limited to a small portion, sometimes involving the whole organ, it is not unreasonable to admit that an inversion should be initiated, as well as completed, after having been commenced.

Rokitansky supposes that paralysis sometimes occurs in the portion of the uterus to which the placenta was attached, and that inversion may be the result of the unequal action. Be this as it may, there can be no doubt that though often the direct result of gross ignorance and violence, it has also happened to the most careful and judicious, not only without their agency, but in spite of their efforts to prevent it. Mr. Crosse remarks that this should be kept in mind, in order to remove every inducement for concealing what has happened, for the truth will, in the end, be equally advantageous both to science and the public.

"Uterine inversion has been mistaken more often in proportion to its frequency than any other malady of its class, or perhaps of any class."

Besides the mistakes in diagnosis acknowledged by Velpeau and other eminent surgeons, Mr. Crosse cites one specimen which had for years been considered an inversion of the uterus, and was put up as such in a pathological museum, but which he demonstrated to be a fibrous polypus, originating just within the cervix, obliterating the os, and at length causing atrophy of the body of the uterus. The existence of the distinctive mark—the circular furrow of uniform depth around its upper portion, together with an unvarying size—will ordinarily serve to distinguish inversion from polypus; but exceptional cases require the aid of the other means of diagnosis. I lately examined a tumor, corresponding in size and uniformity of surface to an inversion, where, owing to an unusually horizontal position of the uterus, the sound could not be passed freely, and it was difficult to say whether at any one point it penetrated more than the depth of an inch. It proved to be a fibrous polypus, with broad attachment within the neck. In general, the physical signs of inversion are positive, and admit of being defined—a consideration of some importance as an element in diagnosis.

In these three cases, the previous history, and the distinctly marked sulcus, left little room for doubt; but it was also ascertained, by pushing upward the vaginal cul de sac, and making a corresponding pressure upon the pubes, that the uterus was not in its place; and by examining through the rectum and vagina, the whole organ could be comprised between the fingers. In one of the cases, also, I was able to trace the course of the left round ligament into the opening of the sac formed by the inversion.

In reference to treatment, it was to be borne in mind that the chief danger arose from exhausting sanguineous or serous discharge attendant on menstruation—that some subjects were able to tolerate inversion without serious inconvenience, either because the menstrual function was not materially disturbed, or that it ceased prematurely, or that they had strength enough to withstand the hemorrhage until they had reached the period of its natural cessation. Some instances of recovery have followed the natural process of sphacelation; but if it occur at all, it would be looked for within the first few days, though in one case, cited by Mr. Radford, it did not occur till six months, and in another, not until six years after delivery. The account of the latter case may be found in the Dict. des Sciences Médicales, xxi, 219. The subject of the inversion, which, from its symptoms, probably protruded externally, was a hard-working woman, and the tumor was much irritated by the contact of urine and unavoidable friction. At the end of six years it increased in size, became livid, was covered with eschars, and was finally detached and thrown off. She recovered and lived three years in good health. On examination after death, the uterus was found wanting, its place being occupied by part of the small intestine. Owing to imperfect cicatrization, it was found that the handle of the scalpel could be passed freely from the cavity of the pelvis, through the neck of the uterus, into the vagina; a condition which was presumed to account for a sensation of cold in the abdomen when she was not carefully protected by clothing.

The probabilities of spontaneous re-inversion would hardly enter into a calculation, though evidence of the fact is well attested. Two cases, especially, have been recorded by Dr. Meigs, which were verified by himself and other gentlemen of eminence, after a most deliberate and careful examination.

The first duty, of course, is to attempt a re-inversion, and to this end full etherization is requisite. We tried this unsuccessfully; and a statement of Velpeau, that he was unable to effect it, after death, shows that it is sometimes impossible. But the fact that reposition has sometimes occurred spontaneously, and the few instances in which it has been accomplished by art, warrant a persevering though cautious trial. We may be fortunate enough to meet that peculiar junction of forces, just balancing between relaxation and contraction, that needs but little aid to be effectual. One thing which is felt to be essential to success, is a fulcrum on which to operate; for the vagina affords a very unsteady resistance, and would be in danger of laceration by a force sufficient to make an impression on the uterus. The upper part may be reached and steadied by one or more fingers through the rectum; but a still more judicious manipulation is that first employed by M. Barrier, who under full etherization introduced the whole hand into the vagina, and having full control of the uterus, successfully restored it by pressing up the fundus with the thumb, while the upper portion was held firmly by the fingers.

The attempts at reposition having failed, we were to consider the expediency of an operation for its removal. The occurrence of spontaneous separation by gangrene would seem to imply that the vitality of the organ is lowered by its unnatural position, and that it would more readily yield to a ligature. In point of fact, the mortality after operations was about one in four; but if even it were still greater, it would certainly be proper to operate if the patient were in immediate danger, or if, judging from the rate at which she has been sinking, we have reason to believe that she will soon have lost the only favorable time for recovery.

The nervous system may become so shattered as to be unable to bear the first shock of the ligature. "A woman, who, for five months, has been exhausted by hemorrhage, pain and want of sleep, entered the hospital at Lyons for removal of what was considered a fibrous polypus. She was represented to be frightfully pale, and subject to frequent fainting. The outcry of the patient on the tightening of the ligature revealed the existence of inversion. It was immediately removed, and she placed in bed, but, notwithstanding every care, died on the fifth day. On examination after death, there was no evidence of peritonitis, or other lesion, and we may fairly presume that the result would have been different if the operation had been done earlier.

The ligature has proved to be the most successful means of operating, but a very important question arises in regard to the mode of applying it—whether the strangulation shall be immediate or gradual. Immediate strangulation has been successfully practised, and on the same principle as for the removal of polypus. The two cases, however, are not analogous. The one is a sensitive, vital organ; the other an insensible growth. Polypus, it is true, has a sensitive covering, prolonged from the part it springs from; but this is superficial, and is annulled by the first constriction of the ligature. On the other hand, not only must the vitality of the uterine walls be destroyed, but adhesion of the peritoneum must be effected in order to efface the cavity.

In each of these cases immediate strangulation was attempted, but the failure of the pulse, and other symptoms of sinking, made it necessary to desist; and throughout the treatment the pressure of the ligature was altered, even three or four times a day, but so as to maintain a degree of constriction that could be tolerated with the aid of opium and ether.

I should have stated that the ligature employed was a small linen cord.

Dr. Hunt, of Buffalo, mentions 14 cases of operation—4 of which were fatal. These were collated in 1853. He does not give the references, but states that "many, perhaps most of his cases, are included in Mr. Crosse's statistics, and that adding the two together we have 12 deaths in 47 operations." The mortality deduced, by Mr. I. G. Forbes, from 27 cases, some of which, no doubt, were referred to by Dr. Hunt, is not materially different.

I subjoin the following, that have been published since 1853:

Dr. Giddings. Charleston Medical Journal, 1854. Entire inversion, of "many years standing." It hung between the thighs, and was of the size of the fœtal head at the term. Removal successfully effected by ligature and incision.

Mr. Teale. London Medical Times and Gazette, 1855. Partial inversion. Removed successfully by double ligatures, five years after the last parturition.

London Lancet, 1855. Sequel to an operation by ligature performed thirty-six years ago. The health had, in the interval, been re-established, and at the autopsy cicatrization was complete.

Mr. Oldham. British and Foreign Review, 1856. Two cases of successful operation by ligature, in one of which it was applied for removal of polypus, but included a portion of the uterus which had been dragged downward.

Since the above, Dr. D. H. Storer has successfully removed, by ligature, an inversion of three years' standing.

Adding to this collection the three cases I have reported, we have nine cases of operation. One of them proved fatal, as I have before remarked, not in consequence of the operation, but on account of ill-timed exertion afterward. Including it, however, we have one fatal and eight successful cases. The inversion was partial in all but one of the cases—that of Dr. Giddings, in which "the incision passed through the walls of the vagina."—*Boston Med. and Surg. Jour.*

